# Astrin: A Key Player in Mitosis and Cancer

**DOI:** 10.3389/fcell.2020.00866

**Published:** 2020-08-28

**Authors:** Zhenguang Ying, Jing Yang, Wei Li, Xia Wang, Zeyao Zhu, Weipeng Jiang, Chunman Li, Ou Sha

**Affiliations:** ^1^Department of Anatomy, Histology and Developmental Biology, School of Basic Medical Sciences, Shenzhen University Health Science Centre, Shenzhen, China; ^2^School of Dentistry, Shenzhen University Health Science Centre, Shenzhen, China

**Keywords:** Astrin, mitosis, kinetochore, microtubules, spindle, cancer

## Abstract

Astrin, which is a spindle-associated protein, was found to be closely related to mitotic spindle formation and maintenance. It interacts with other spindle-related proteins to play a key role in maintaining the attachment of the kinetochore-microtubule and integrity of centrosomes and promoting the centriole duplication. In addition, Astrin was quite recently found to be abnormally highly expressed in a variety of cancers. Astrin promotes the development of cancer by participating in various molecular pathways and is considered as a potential prognostic and survival predictor.

## Introduction

Cell division represents the most important life activity of cells and it forms the basis of growth, reproduction, and genetic inheritance of living organisms. Mitosis is the most common form of cell division in higher animals. Only by relying on mitosis can the cell ensure accurate replication of its genome and fair division to its daughter cells.

Mitosis is usually divided into five key phases that are defined as: prophase, prometaphase, metaphase, anaphase, and telophase, which are characterized by chromosome condensation, chromosome alignment to the metaphase plate, chromosome segregation to an opposite pole, and cytokinesis, respectively (Schatten, 2013). Normal mitosis requires the formation of the correct bipolar spindle at metaphase, with microtubules (MTs) from both poles attached to kinetochores (KTs) of the sister chromosomes. This process enables chromosomal alignment at the metaphase plate, and ensures that the chromosomes divide equally to two daughter cells in anaphase and telophase (Compton, 2000; Uhlmann, 2001).

The kinetochore-microtubule (KT-MT) dynamic plays a particularly important role here. Misattachment of the KT-MT complex leads to spindle assembly checkpoint (SAC) activation and arrests the cells from advancing to the onset of anaphase. Once the correct KT-MT attachments are formed, and the chromosomes are aligned on the metaphase plate, the cohesin between the sister chromatids is cleaved by separase and the cell will enter anaphase (Foley and Kapoor, 2013; Musacchio and Desai, 2017). Incorrect chromosomal segregation caused by spindle defects was linked to tumor development (Silkworth et al., 2009).

**FIGURE 1:**
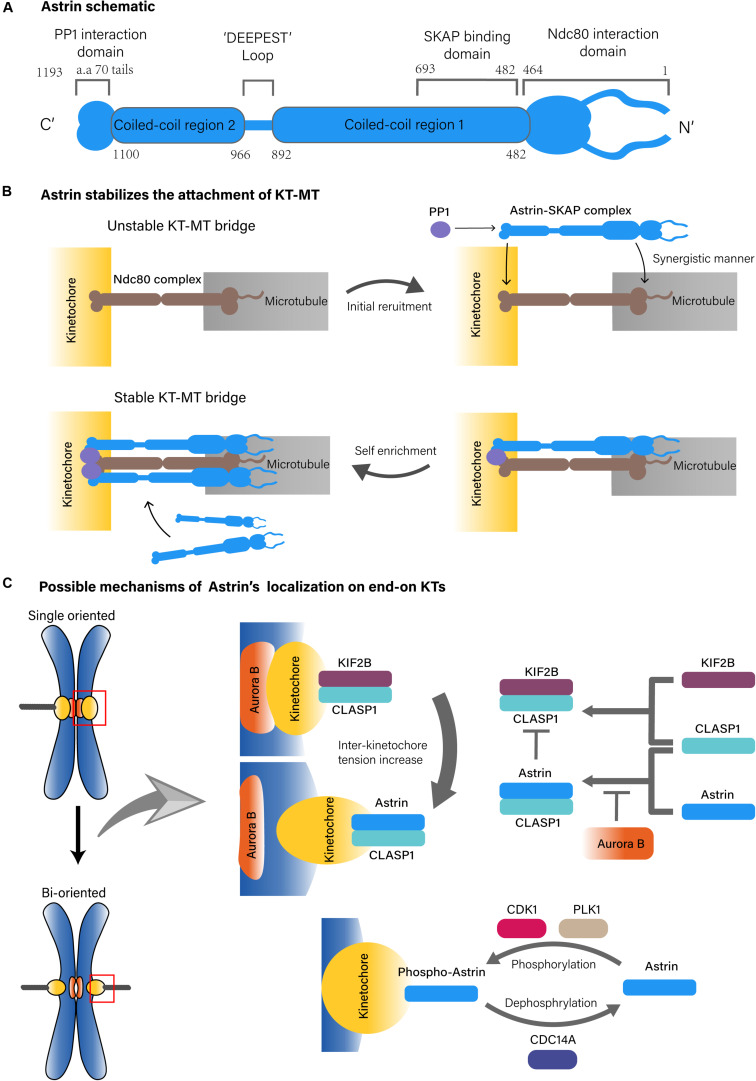
Possible structure of Astrin complex and KT-MT localization mechanisms of Astrin. **(A)** Astrin schematic. **(B)** Astrin stabilizes the attachment of KT-MT. **(C)** Possible mechanisms of Astrin’s localization on end-on KTs.

Many associated proteins are involved in KT-MT attachment, such as nuclear division cycle 80 (Ndc80) complex, Aurora kinase B (AURKB) and others (Cheeseman and Desai, 2008). Astrin, also known as sperm associated antigen 5 (SPAG5) or mitotic spindle associated protein p126 (MAP126), was identified as a MT-associated protein with a coiled-coil structure (Figure 1A) (Chang et al., 2001; Mack and Compton, 2001). Many studies have found that Astrin is one of the key molecules in cellular mitosis, especially in the context of spindle formation and chromosomes alignment (Mack and Compton, 2001; Gruber, 2002; Thein et al., 2007).

In this review, we have highlighted the latest findings regarding the role of Astrin in KT-MT attachment during mitosis, and summarized the functions in centriole duplication and centrosome integrity. In addition, we have briefly outlined recent reports of Astrin expression in cancer and its relevant signaling pathways.

## The Dynamic Localization of Astrin in Mitosis

In cultured mammalian cells, Astrin was first found to diffuse in the cytoplasm and colocalize with pericentriolar material (PCM1) at the centrosome during interphase. As the cells entered mitosis and the spindle formed, it became evident on the mitotic spindle and KTs of aligned chromosomes (Mack and Compton, 2001; Kodani et al., 2015). The knockdown of Astrin expression lead to abnormal multipolar spindle formation, defects in chromosome alignment and segregation (Gruber, 2002; Thein et al., 2007). These findings suggest that Astrin acts as a critical player in mitosis.

### Astrin Plays a Key Role as a “Lock” to Strengthen Stability of End-on KT-MT Attachments

In KT-MT attachment, spindle MTs attach end-on to KTs, a number of conserved protein complexes on chromosomes. The Ndc80 complex is one of the core kinetochore components that plays a primary role in the attachment of MTs to chromosomes (Cheeseman and Desai, 2008; Ustinov et al., 2020). Astrin, which forms a complex with small kinetochore associated protein (SKAP) and other partners that include MYC binding protein (MYCBP) and dynein light chain (LC8), also play key roles in stabilizing KT-MT attachment (Schmidt et al., 2010; Dunsch et al., 2011; Kern et al., 2017).

On the MT side, the Astrin-SKAP complex directly binds to the plus end of MTs through the plus end tracking activity of SKAP, in which the N-terminus of Astrin containing the SKAP binding domain (aa 482–693) is necessary (Dunsch et al., 2011; Kern et al., 2016, 2017). However, the MT binding of the Astrin-SKAP complex is more complex since it also interacts with the Ndc80 complex, in which the N-terminus of Astrin (aa 1–464) and the MT interface of the Ndc80 (aa 1–201) are necessary. Moreover, the Astrin-SKAP complex and the Ndc80 complex can bind MTs simultaneously to co-assemble an integrated interface. This suggests that the Astrin-SKAP complex and the Ndc80 complex jointly form a robust binding to MTs (Figure 1B; Kern et al., 2017).

On the KT side, the localization of the Astrin-SKAP complex requires Astrin’s C-terminus (Kern et al., 2017). Astrin interacts with phosphatase 1 (PP1) through its C-terminal structural motif (70 aa tail), which might deliver PP1 in close proximity to the C-terminus of the Ndc80. The KT recruits PP1 in turn also promotes the enrichment of Astrin at end-on KTs. Although the absence of Astrin’s PP1 interaction motif does not affect its MT localization, it does reduce the localization of Astrin on KTs, which further results in fewer end-on KTs and reduced microtubule-mediated pulling, and promotes delayed onset of anaphase (Conti et al., 2019). These observations suggest that Astrin-PP1 acts as a “lock” to strengthen the stability of the KT-MT bridges that were initially generated by the Ndc80 complex at end-on KTs, which enables KTs to withstand the pulling from the spindle MTs (Kern et al., 2017; Conti et al., 2019). In addition, the interaction between Astrin and PP1 stabilizes end-on attachment under dynamic regulation because if PP1 artificially interacts with Astrin constitutively, the dynamic stability of the end-on attachment will be disrupted and might delay chromosomal alignment and anaphase onset, it will promote defective chromosome segregation (Figure 1B; Conti et al., 2019).

The timing of Astrin localization on end-on KTs relies on more complex mechanisms. One of these mechanisms is based on the tension sensing regulation of Aurora-B, an important serine/threonine kinase that is localized to the chromosome centromere during prometaphase and metaphase. As a molecular switch, Aurora B recognizes an incorrect KT-MT attachment, and does so by sensing the tension across the centromere to regulate the biorientation of the chromosome (Ducat and Zheng, 2004; Liu et al., 2009). During mitosis, Aurora B mediated phosphorylation can antagonize KT localization of Astrin. Then, as the mitotic chromosomes are gradually aligned under tension, Aurora B kinase is separated from the KT substrates, which causes recruitment of Astrin on bi-oriented KTs (Schmidt et al., 2010; Shrestha et al., 2017). This process may also include kinesin family member 2B (KIF2B) and cytoplasmic linker associated protein 1 (CLASP1). In the prophase of mitosis, Aurora B promotes the recruitment of the KIF2B-CLASP1 complex on KTs due to a low inter-KT tension. However, with progression toward metaphase and chromosomes to be bi-oriented, KIF2B-CLASP1 is replaced by Astrin-CLASP1 due to an increased inter-KT tension and a decreased spatial level of Aurora B, which would allow recruitment of Astrin on end-on KTs (Figure 1C; Manning et al., 2010).

In fact, both Astrin-PP1 and Aurora B regulate end-on attachment stability. Inhibition of Aurora B does not stabilize the end-on attachment in the absence of Astrin interacting with the PP1 structural motif. This suggest that the end-on attachment stabilization mediated by inhibition of Aurora B, depends on the Astrin-PP1 interaction. Conversely, under conditions of the presence of Aurora B activity, the interaction of Astrin-PP1 cannot stabilize the end-on attachments (Conti et al., 2019).

In conclusion, Astrin affects the rapid and selective stability of KT-MT attachments, and does so via two key steps. First, the reduction in Aurora B allows the initial recruitment of Astrin on end-on KTs. Then, the interaction of Astrin-PP1, which is in close proximity to the C-terminus of the Ndc80 complex, promotes Astrin enrichment at end-on KTs. Due to synergistic microtubule binding of the Astrin-SKAP complex and the Ndc80 complex, the stability of the attachments is enhanced rapidly and greatly at end-on KTs (Kern et al., 2017; Conti et al., 2019). However, it must be mentioned that the KT-MT regulatory mechanism based on sensing tension remains a hypothetical paradigm. There is also at least one other study that believes that the tension on the KTs is insufficient to enable Aurora B to recognize the state of the KT-MT attachment (de Regt et al., 2018). Moreover, Conti’s work found that Astrin can be recruited to KT before bi-orientation of the chromosomes, which means that the selective KT localization mechanism of Astrin-PP1, might not rely on the tension recognition of Aurora B (Conti et al., 2019). In addition, knocking out of Astrin did not affect the survival or fertility of mice, suggesting that there may be a compensatory mechanism to maintain the stability of KT-MT instead of Astrin (Xue et al., 2002).

The KT localization of Astrin is also regulated by mitotic kinases including polo like kinase 1 (PLK1), and cyclin dependent kinase (CDK) 1. In this regulatory pathway, PLK1 actives CDK1 to phosphorylate Astrin at Ser135 and Ser249, which targets phospho-Astrin to KTs. In contrast, the phosphorylase cell division cycle (CDC) 14A dephosphorylates Astrin and negatively regulates Astrin localization on KT (Figure 1C). This indicates that the dynamic kinetochore localization of Astrin is also regulated by mitotic kinases (Chung et al., 2016).

### Astrin Localizes to Centrosomes to Promote Centriole Replication During Interphase, and Maintain the Integrity of Centrosomes During Mitosis

In addition to the role in KT-MT attachments, Astrin also localizes to centrosomes with PCM1 throughout the cell cycle, and is involved in centriole duplication (Kodani et al., 2015). During interphase, Astrin interacts with centrosomal protein (CEP) 72 and primary microcephaly (MCPH) associated protein CDK5RAP2 (CDK5 regulatory subunit associated protein 2). Both Astrin and CEP72 are required for the centrosome localization of CDK5RAP2. The centrosomal localization of CDK5RAP2 further causes sequential recruitments of other CDK5RAP2 dependent MCPH associated proteins like CEP152, CEP63 and etc. to assemble a hierarchical MCPH complex at the centrosome. They promote centrosomal localization of CDK2, which is a key cyclin dependent kinase playing roles in centriole duplication (Matsumoto et al., 1999; Kodani et al., 2015). Depletion of Astrin not only resulted in reduced CDK5RAP2 localization on the centrosome and dampened its stability, but it also reduced centrosomal localization of CDK2. This further decreased the number of centrioles at the S-Phase of the cell cycle, which is similar to the effect of depleting CDK5RAP2 (Kodani et al., 2015).

Additionally, Astrin localizes to the spindle pole during prometaphase to metaphase (Mack and Compton, 2001). In fact, the presence of Astrin is necessary for the integrity of centrosomes during mitosis. Knockdown of Astrin results in centriole disengagement during metaphase, which causes the formation of a multipolar spindle with a single centriole at each pole. This might in part be due to Astrin being involved in blocking the activity of separase, which actives segregation of sister chromatids at anaphase, and disengagement of centriole at the end of mitosis (Uhlmann, 2001; Tsou and Stearns, 2006; Thein et al., 2007).

### Astrin Is Downregulated at End of Mitosis

At telophase, Astrin shows localization to intercellular bridge microtubules (ICBMTs) and its down-regulation is essential for normal cytokinesis (Mack and Compton, 2001; Gholkar et al., 2016). In mitosis, the ubiquitin ligase midline 2 (MID2) is involved in the regulation of MT stability and organization, and participates in binding proteins to MTs (Suzuki et al., 2010). It negatively regulates the level of Astrin by ubiquitination of the Lysine 409 site of Astrin at intercellular bridge MTs. Expressing the K409A mutant Astrin can cause cytokinesis defects, the possible reason for this is that the mutant Astrin lacks ubiquitination sites and cannot be degraded by ubiquitination through MID2, which results in accumulated Astrin at ICBMTs and two daughter cells that cannot be completely disconnected. These results suggest that Astrin might stabilize ICBMTs, and that Astrin needs to be ubiquitinated at telophase to disengage MTs between the two daughter cells (Gholkar et al., 2016).

### Other Molecules Affecting the Spindle Localization of Astrin

In addition to the aforementioned discussed mechanisms, Astrin’s spindle localization may require the interplay of other functional molecules including glycogen synthase kinase 3 beta (GSK3B) (Cheng et al., 2008). GSK3 is a key protein kinase that coordinates many cellular functions, including cell division and MT dynamics (Cohen and Frame, 2001). Cheng et al. (2008) found that GSK3B phosphorylated Astrin at Thr-111, Thr-937, and Ser-974/Thr-978 during mitosis. If GSK3B activity is inhibited, Astrin will lose its spindle localization during mitosis, which might provoke abnormal spindle structures. This indicates that GSK3B-mediated phosphorylation is critical for proper Astrin spindle localization.

The localization of Astrin also requires the nuclear mitotic apparatus (NuMA) protein that is crucial in assembling the spindle and shows mitotic spindle localization. First, Astrin is necessary for spindle localization of NuMA. In turn, NuMA also contributes to the recruitment of MTs and spindle pole localization of Astrin. It was also found that Astrin was recruited to MTs during interphase through direct interactions between its C-terminal domain and the C-terminal domain of NuMA. Interestingly, overexpression of the NuMA C-terminus enabled recruitment of Astrin to spindle poles, which was alternatively decreased at KTs. This coordination of Astrin, suggests that its localization between spindle poles and KTs may be balanced by NuMA (Chu et al., 2016).

In addition to what is known about Aurora B, it should be noted that Astrin also interacts with Aurora kinase A (AURKA), which is another member of the mitotic-regulated serine/threonine kinase family (Bischoff and Plowman, 1999; Du et al., 2008; Chiu et al., 2014). Interestingly, the relationship between Astrin and Aurora A appears to have two distinct characteristics. First, Astrin acts as an upstream regulator of Aurora A, enabling the localization of Aurora A to the spindle (Du et al., 2008). Second, Astrin is the phosphorylated substrate of Aurora A, and Aurora-A is essential for Astrin to regulate the activity of separase. Further, the Aurora A phosphorylation site of Astrin is important for the association of Astrin with additional interacting proteins including CLASP1, LC8, SKAP, and others (Chiu et al., 2014). In short, the relationship between Astrin and Aurora A warrants further study.

## The Role of Astrin in Cancer

Genetic instability is the most common characteristic of human tumor formation. In tumor cells, aneuploid or abnormal chromosomes are the result of abnormal mitosis due to a variety of reasons including multipolar mitotic spindles, defects in KT-MT attachment, chromosome segregation defects, and others (Kops et al., 2005). Astrin, which is an essential mitotic protein has been found to display abnormally high expression and is associated with tumor prognoses in many cancers (Abdel-Fatah et al., 2016; Liu H. et al., 2018; Liu J. Y. et al., 2018; Liu et al., 2019; Zhang et al., 2020). Current research has found that Astrin plays a key role in activating many signaling pathways that are known to be associated with cancer, including the WNT/β-catenin and PI3K/AKT signaling pathways (Yang et al., 2018; Jiang et al., 2019). This informs us that the important mitotic protein Astrin may instigate an unusual role in cancer development.

### Astrin in Breast Cancer

The high expression of Astrin was significantly associated with the development and prognosis of breast cancer (Abdel-Fatah et al., 2016). Specifically, copy number mutations of Astrin and its high transcriptional and protein expression were linked to a shorter survival period and a poorer prognosis in breast cancer patients. High expression of Astrin was associated with both malignancy and invasiveness of breast cancer, and often led to a higher risk of recurrence and lymphatic metastasis (Jiang et al., 2019; Li et al., 2019).

Overexpressed Astrin also increased the expression of Wnt family member 3 (WNT3) and β-catenin, activating the WNT/β-catenin signaling pathway (Jiang et al., 2019). In addition, Astrin was found to interact with MYCBP to promote the transcriptional activity of *c-MYC*, which could promote cell proliferation via upregulating c-MYC targeted genes including *CDC20*, *CDC25C*, breast-cancer susceptibility gene *(BRCA)* 1, *and BRCA2* in triple-negative breast cancer (Li et al., 2019).

### Astrin in Hepatocellular Carcinoma

Astrin also plays a cancer-promoting role in hepatocellular carcinoma (HCC) (Liu H. et al., 2018; Yang et al., 2018). It was found that overexpressed Astrin improved the stability of β-catenin by inhibiting the ubiquitination of β-catenin through ubiquitin proteasome system, which induces expression of scavenger receptor class A member 5 (SCARA5), a new member of the scavenger receptor family, and activates its role in suppressing disease progression and metastasis in cancer (Armengol et al., 2013; Liu H. et al., 2018). In addition, Astrin can interact with CEP55 to enable activation of the PI3K/AKT signaling pathway, which is a most commonly activated signaling pathway in HCC (Xia and Xu, 2015; Yang et al., 2018). Those mechanisms eventually lead to HCC cellular proliferation.

### Astrin in Other Cancers and Possible Inhibitor

The abnormal expression of Astrin in a variety of tumors has been reported. Astrin was also associated with tumor activity and prognosis in cervical, prostate, lung, bladder, and gastric cancer (Välk et al., 2010; Yuan et al., 2014; Zhang et al., 2016, 2020; Liu J. Y. et al., 2018; Song et al., 2018; Liu et al., 2019; Wang et al., 2019). Herein we have summarized cancers that were reported to display highly expressed Astrin, and the involved signaling pathways (Table 1).

**TABLE 1:** The expression of Astrin and its effects, involved downstream signaling pathways and possible upstream suppressor about Astrin in each type of cancer.

**Types of cancer**	**Expression of Astrin and its effects**	**Downstream pathways**		**Possible upstream suppressor**	**References**
			**Up/Down regulated**		
Breast cancer/Triple negative breast cancer	Upregulated and associated with short survival, high combination cytotoxic chemotherapy sensitivity	Wnt/β-catenin	Up	N.A.	Jiang et al., 2019
		MYCBP/c-MYC	Up		Li et al., 2019
Hepatocellular carcinoma	Upregulated and associated with worse survival	β-catenin/SCARA5	Up	miR-363-3p	Liu H. et al., 2018
		CEP55/PI3K/AKT	Up		Yang et al., 2018
Cervical cancer	Upregulated and associated with poor prognosis, and affected taxol sensitivity by different drug concentrations	mTOR pathway	Alter	miR-367-3p	Yuan et al., 2014; Yang et al., 2020
Prostate cancer	Upregulated and associated with higher grade and poor prognosis	N.A.		miR-539	Zhang et al., 2016
Bladder urothelial cancer	Upregulated and associated with worse survival, resistance to chemotherapy	AKT-mTOR/WNT3 pathways	Up	N.A.	Liu J. Y. et al., 2018
Non-smallcell lung cancer	Upregulated and associated with poor prognosis	N.A.		*p53-p21* axis	Välk et al., 2010; Wang et al., 2019
				miR-1179	Song et al., 2018
Gastric cancer	Upregulated and associated with poor prognosis	Wnt/β-catenin/Survivin	Up	N.A.	Liu et al., 2019
Ovarian cancer	Upregulated and associated with poor prognosis	N.A.		N.A.	Zhang et al., 2020

Astrin expression also affects the sensitivity of cancer cells to chemotherapy. In breast cancer, high Astrin expression is associated with a higher sensitivity of combination cytotoxic chemotherapy; however, Astrin knockdown also increased sensitivity to the poly ADP-ribose polymerase inhibitor Olaparib. In cervical cancer, Astrin can differentially regulate the mammalian target of rapamycin (mTOR) pathway in the process of tumor cell resistance to chemotherapeutic drugs including taxol (Yuan et al., 2014). In fact, Astrin was reported to behave as a negative regulator of mTOR complex 1 (mTORC1) by competing with mTOR to bind raptor under conditions of oxidative stress (Thedieck et al., 2013).

Furthermore, Astrin has been found to be negatively regulated by miRNAs. For example miR-363-3p could inhibit the expression of Astrin in HCC by binding the 3′UTR of Astrin mRNA in HCC cells (Yang et al., 2018). Similar reports have also shown the importance of miR-539, miR-1179, and miR-367-3p in prostate, lung and cervical cancers (Zhang et al., 2016; Song et al., 2018; Yang et al., 2020). These miRNAs can directly target Astrin and inhibit its expression with the net result of dampening the proliferation and invasiveness of cancer cells. This suggests that the use of miRNA to inhibit Astrin expression might be licensed as a potential cancer therapy.

## Conclusion

As a spindle associated protein, Astrin plays a critical role in mitosis. It is involved in the formation of spindle structures, participates in dynamic KT-MT attachments, ensuring the correct alignment and segregation of chromosomes. In addition, Astrin is associated with tumorigenesis. It may not only serve as a prognostic predictor of various tumors, but it might also serve as a potential therapeutic target in clinical practice in the future. However, the underlying mechanism of how Astrin promotes tumorigenesis remains unclear, and few studies have elucidated the mechanism of Astrin’s pro-tumor activity in terms of its role in mitosis. With the deepening of research on the functional role of Astrin, the discovery of more relevant molecular mechanisms might reveal novel insights and understanding from carefully targeted research of cancer and other diseases.

## Author Contributions

ZY contributed to the conceptual idea, reviewed, analyzed the literature, and wrote the manuscript. JY and WL assisted in compiling background material. XW, ZZ, and WJ edited the manuscript and provided suggestions for the revision of the manuscript. CL and OS provided the conceptual idea, analyzed the literature, reviewed, and edited the manuscript. All authors contributed to the article and approved the submitted version.

## Conflict of Interest

The authors declare that the research was conducted in the absence of any commercial or financial relationships that could be construed as a potential conflict of interest.
